# Predicting of the Coronavirus Disease 2019 (COVID-19) Epidemic Using Estimation of Parameters in the Logistic Growth Model

**DOI:** 10.3390/idr13020046

**Published:** 2021-05-24

**Authors:** Agus Kartono, Setyanto Tri Wahyudi, Ardian Arif Setiawan, Irmansyah Sofian

**Affiliations:** Department of Physics, Faculty of Mathematical and Natural Sciences, IPB University (Bogor Agricultural University), Jalan Meranti, Building Wing S, 2nd Floor, Kampus IPB Dramaga, Bogor 16680, Indonesia; stwahyudi@apps.ipb.ac.id (S.T.W.); aarif@apps.ipb.ac.id (A.A.S.); irmansyah@apps.ipb.ac.id (I.S.)

**Keywords:** COVID-19, epidemic, least-squares, logistic model, prediction

## Abstract

The COVID-19 pandemic was impacting the health and economy around the world. All countries have taken measures to control the spread of the epidemic. Because it is not known when the epidemic will end in several countries, then the prediction of the COVID-19 pandemic is a very important challenge. This study has predicted the temporal evolution of the COVID-19 pandemic in several countries using the logistic growth model. This model has analyzed several countries to describe the epidemic situation of these countries. The time interval of the actual data used as a comparison with the prediction results of this model was starting in the firstly confirmed COVID-19 cases to December 2020. This study examined an approach to the complexity spread of the COVID-19 pandemic using the logistic growth model formed from an ordinary differential equation. This model described the time-dependent population growth rate characterized by the three parameters of the analytical solution. The non-linear least-squares method was used to estimate the three parameters. These parameters described the rate growth constant of infected cases and the total number of confirmed cases in the final phase of the epidemic. This model is applied to the spread of the COVID-19 pandemic in several countries. The prediction results show the spread dynamics of COVID-19 infected cases which are characterized by time-dependent dynamics. In this study, the proposed model provides estimates for the model parameters that are good for predicting the COVID-19 pandemic because they correspond to actual data for all analyzed countries. It is based on the coefficient of determination, *R*^2^, and the *R*^2^ value of more than 95% which is obtained from the non-linear curves for all analyzed countries. It shows that this model has the potential to contribute to better public health policy-making in the prevention of the COVID-19 pandemic.

## 1. Introduction

Infectious diseases often have a negative impact so it remains a challenge for medical and public health authorities. Therefore, controlling an outbreak of infectious diseases requires immediate treatment, however, identifying and characterizing an outbreak in real-time is still a challenge. Knowing the characteristics of an outbreak of infectious diseases is necessary for decision-making quickly and concrete in controlling the infectious disease. By knowing the prediction of an infectious disease epidemic in the short term so evaluation of the impact caused by this disease can be reviewed from the preventive measures for this disease [1]. Therefore, a prediction model is needed to obtain a prediction result of the epidemic to contain the spread rate of the disease. The real-time mathematical model for analyzing an epidemic is an important prevention method. Due to the limited availability of public information on the characteristics of new epidemics and pathogens, it will pose obstacles to the creation of reliable and credible models during public health emergencies [2,3].

The accuracy of a model is dependent highly on the data characteristics of the ongoing epidemic. Some epidemic curves show multiple turning points (such as peaks and valleys) during the early stages of the outbreak, probably due to stochastic variations and the random nature of disease spread. There are also changes in surveillance methods, case definitions, and turning points which can also indicate the turning times in which the epidemic is in the process of transitioning from an exponential growth process to a decline in growth rate. However, it can also identify the effects of disease control programs, peak-phase waves of infection or the natural slowdown in the growth of susceptible individuals are resulting from infection. In each epidemic, there are points in time corresponding to the phases of a particular outbreak that can be modeled precisely and accurately, but there are also phases outside the outbreak that are likely to occur but this can be anticipated. Therefore, the prediction of turning points and the identification of different phases or waves of an epidemic are very important for designing and evaluating various strategies for controlling an epidemic [4,5].

The COVID-19 pandemic is still ongoing in several countries, so it is still a new challenge for medical and public health authorities because this epidemic is a threat to the whole world. This epidemic must be well controlled, therefore the early step that must be started is to obtain accurate information about the prediction of the final time of this epidemic. Although the prediction process will be much more difficult because it is the result of a complex series of actions and interactions between a population and a disease transmitter. In addition, other factors that influence predictions are the effect of quarantine, suspect testing policies, and vaccinations, as these have an impact on the future circulation of the virus. Today, the first wave of the COVID-19 pandemic has reached its peak in some countries, while in other countries it is still in various stages of spread. The countries that have managed to control this epidemic are trying to avoid a second wave of the COVID-19 pandemic, while the countries that are still experiencing this epidemic or are currently an outbreak are trying to contain the spread of this outbreak in society.

It is well known that the logistic growth model can capture the actual data evolution of many epidemic events that have occurred. This model can accurately describe an epidemic in a closed population with a limited population and also follows a deterministic model solution [6,7,8,9,10,11,12,13]. Therefore, this study aims to propose the logistic growth model as a new approach to epidemic models that will be compared with actual data of the reported cumulative infected cases. This study proposes the phenomenological model to match the observation cumulative data of the COVID-19 infected cases so that this model can describe the phases of the epidemic sequentially. This model is also a predictive method for estimating the final time and total size of the final epidemic in several countries. This model is used to describe the actual data on the phases of the COVID-19 pandemic. The accuracy of the correlation relationship between the prediction results of this model and the actual data is based on the coefficient of determination, *R*^2^ [14]. The *R*^2^ value will be calculated as a product of the match between the prediction results and the actual data of the number of infected cases reported to the public.

This paper is structured as follows: Section 2 describes the logistic growth model proposed in this study. In this section, this model is described in detail. Section 3 describes the estimation method of model parameters using the non-linear least squares. A discussion of the prediction results of the model in this study is given in Section 3. Section 4 provides conclusions and the main contribution of this study.

## 2. The Logistic Growth Model

The logistic growth equation with positive parameters has been widely applied in economics and biology. Although this equation does not allow for adjustment to the actual data (quantitatively), because it is still a rough estimate (qualitatively) and does not correspond to the stochastic elements. However, the estimation of this equation can be solved without the need for complicated techniques and only uses basic regression methods. Even though it only needs a simple estimation method, some difficulties still arise to solve the estimation that arises which may limit the practical application of this equation [15]. Therefore, choosing a suitable estimation method is often the right choice.

The development of a logistic growth model by Verhulst [16] which describes the growth rate equation of a population is defined as follows:

(1) $$\frac{dN\left(t\right)}{dt}=rN\left(t\right)\left(1-\frac{N\left(t\right)}{K}\right)\;,$$

the constant *r* defines the intrinsic growth rate of the population while the constant *K* defines the carrying capacity of the environment. Some characteristics of the ordinary differential equation of the logistic growth model that need to be considered are when the *N* value is less than the *K* value (*N* << *K*), then:

(2) $$\frac{dN\left(t\right)}{dt}\approx rN\left(t\right)\;,$$

this means that as long as the *N* value is still small, the population will grow exponentially. Furthermore, if the *N* value approaches the *K* value (*N* → *K*), then the population growth rate is close to zero (*dN*/*dt* → 0) which will result in the population growth starting to stop gradually. So, it can be concluded that the *N* value is equal to *K* (*N* = *K*) or is saturated, this means that the carrying capacity described above has reached the maximum value.

In Equation (1), this equation is separated according to the variables then followed by algebraic rearrangement to produce a partial differential equation. Furthermore, this equation is integrated exponentially with algebraic manipulation will produce the following equation:

(3) $$N\left(t\right)=\frac{K}{1+A\;\exp\left(-r\left(t\right)\right)}\;,$$

where

(4) $$A=\frac{K-N\left(0\right)}{N\left(0\right)}$$

The curve shape of Equation (3) is defined as sigmoid-shaped or *S*-shaped with a slope symmetrical at the inflection point (peak time) for the value of *N* = *K*/2 which is defined as follows:

(5) $$t_{p}=\frac{\log\left(A\right)}{r}\;,$$

and Equation (3) is characterized by two limitations as follows:

$$\underset{t\to +\infty }{\lim}N\left(t\right)=K,$$

and

$$\underset{t\to -\infty }{\lim}N\left(t\right)=0\;.$$

This shows that with increasing time, the population will reach its carrying capacity.

The peak time of the epidemic is the turning point in the total number of cumulative infected cases. After this peak time, the time evolution of the epidemic cannot be approximated by a simple exponential growth curve, since during this phase of the epidemic there is no more multiplication of infected cases in this time evolution. In this phase, the daily number of infected cases will decrease.

In cases with a value of *r* > 0, the results of the growth curves will have a sigmoid shape, so that there is an asymptotic bearing capacity based on Equation (3). In a case with a value of *r* = 0, there is no intrinsic growth rate, so the population will always be constantly equal to the initial value *N*(0). In general, biologists and ecologists of the population are particularly interested in cases where a value of *r* > 0, so this study always assumes this case. Furthermore, the population size *N* can always be assumed to be positive.

In this study, the variable *N* represents the number of the COVID-19 infected cases at a certain time *t*, the parameter *r* represents the growth rate of the COVID-19 cases infected, while the parameter *K* represents the total number of the COVID-19 infected cases cumulatively in the final phase of the epidemic. Therefore, the procedure to estimate the three parameters (*K*, *A*, and *r*) of the model is given by Equation (3) is still difficult, this study will use a non-linear regression method which will be explained in detail in the next section.

## 3. Parameters Estimation of the Logistic Growth Model

In Equation (3), the analytical solution of the logistic growth model consists of three parameters, namely *K*, *A*, and *r*. Furthermore, these parameters will be estimated using a non-linear regression method with the initial input of the actual data set [17]. This approach assumes that the daily cumulative number of infected cases, *M*(*t_i_*), is entered as a data set, {*t_i_*, *M_i_*}, where *t_i_* and *i* represent the index of time and day. The estimated method of the parameter values (*K*, *A*, and *r*) use function:

(6) $$s^{2}=\frac{1}{n}\overset{n}{\underset{i=1}{\sum }}{\left[M_{i}-N\left(t_{i}\right)\right]}^{2}\;,$$

*n* represents the total number of days. The first derivative of Equation (6) guides the determination for the estimation of each parameter is given by:

(7) $$\frac{\partial s}{\partial K}=-\frac{2}{n}\overset{n}{\underset{i=1}{\sum }}\left[M_{i}-N\left(t_{i}\right)\right]\frac{\partial N\left(t_{i}\right)}{\partial K}\;,$$

(8) $$\frac{\partial s}{\partial r}=-\frac{2}{n}\overset{n}{\underset{i=1}{\sum }}\left[M_{i}-N\left(t_{i}\right)\right]\frac{\partial N\left(t_{i}\right)}{\partial r}\;,$$

(9) $$\frac{\partial s}{\partial A}=-\frac{2}{n}\overset{n}{\underset{i=1}{\sum }}\left[M_{i}-N\left(t_{i}\right)\right]\frac{\partial N\left(t_{i}\right)}{\partial A}\;,$$

where

(10) $$\frac{\partial N}{\partial K}=\frac{1}{1+Ae^{-rt}}\;,$$

(11) $$\frac{\partial N}{\partial r}=\frac{rKAe^{-rt}}{{\left(1+Ae^{-rt}\right)}^{2}}\;,$$

(12) $$\frac{\partial N}{\partial A}=-\frac{Ke^{-rt}}{{\left(1+Ae^{-rt}\right)}^{2}}\;.$$

The procedure of this method requires the initial input to estimate the three parameters when *t* = 0: *K*(0), *r*(0), and *A*(0). The corresponding initial input for the three parameters is obtained by selecting the three initial estimates from some actual data: *M_k−_*_2*m*_, *M_k−m_*, and *M_k_* where *k* ≈ *n* and *m* ≈ *n*/2. Based on the initial input of the actual data, the equations of the three parameters can be given as follows:

(13) $$K\left(0\right)\approx \frac{M_{k-m}\left(M_{k-2m}M_{k-m}-2M_{k-2m}M_{k}+M_{k-m}M_{k}\right)}{M_{k-m}^{2}-M_{k}M_{k-2m}}$$

(14) $$A\left(0\right)\approx \frac{\left(M_{k}-M_{k-m}\right)\left(M_{k-m}-M_{k-2m}\right)}{M_{k-m}^{2}-M_{k}M_{k-2m}}{\left[\frac{M_{k}\left(M_{k-m}-M_{k-2m}\right)}{M_{k-2m}\left(M_{k}-M_{k-m}\right)}\right]}^{\left(k-m\right)/m}$$

(15) $$r\left(0\right)\approx \frac{1}{m}\log\left[\frac{M_{k}\left(M_{k-m}-M_{k-2m}\right)}{M_{k-2m}\left(M_{k}-M_{k-m}\right)}\right]\;,$$

the input characteristic of the actual data for the initial approximation requires that all parameters are positive and the value of *K* must be greater than the value of *M_k_* (*K* > *M_k_*). However, in some cases, the data from the initial approach already has very good values so that no exact value is needed to minimize the variance given by Equation (6). Therefore, the steepest descent procedure was applied as a way to minimize the variance in Equation (6).

The coefficient of determination, *R*^2^, is used to analyze and evaluate the differences between the first variable and the second variable. In general, the measure of *R*^2^ value is defined in the range of 0 to 1, or in the percent range, which is 0% to 100%. The measure of the *R*^2^ value can be considered as a description of how many actual data points fit in the line result of the model formed by the regression equation. The higher the coefficient of determination, the higher the percentage of actual data points that the line of the model crosses when the line is plotted on the data. If the coefficient is 0.80, it means that 80% of the actual data points are in the regression line. A higher coefficient is an indicator of better goodness of fit for observation. The coefficient of determination, *R*^2^, has also the ability to find possible predictable outcomes in the future. If more actual data is added, the coefficient will show the probability of the new points corresponding to the regression line of the proposed model.

A data set consists of *N* actual data marked *y*_1_, …, *y_N_*, collectively known as *y_i_* or as the vector *y* = [*y*_1_, …, *y_N_*]. Each of these actual data relates to the simulation results of the proposed model or prediction model, which is marked *f*_1_, …, *f_N_* or as the vector *f* = [*f*_1_, …, *f_N_*]. If $\bar{y}$ is the mean of the observed data can be defined as follows:

(16) $$\bar{y}=\frac{1}{N}\overset{N}{\underset{i=1}{\sum }}{\left(y_{i}\right)}^{2}\;,$$

then the total sum of the squares of the actual data variance can be defined as follows:

(17) $$SS_{tot}=\overset{N}{\underset{i=1}{\sum }}{\left(y_{i}-\bar{y}\right)}^{2}\;,$$

and the sum of the squares of the residuals or commonly referred to as the sum of the squares of the residuals can be defined as follows:

(18) $$SS_{res}=\overset{N}{\underset{i=1}{\sum }}{\left(f_{i}-y_{i}\right)}^{2}\;,$$

thus, the most common definition of the coefficient of determination (*R*^2^) can be declared as follows:

(19) $$R^{2}=1-\frac{SS_{res}}{SS_{tot}}\;.$$

## 4. Results and Discussion

This study will analyze the COVID-19 pandemic in several countries based on data of the cumulative infected cases collected from actual data reported by WHO from the first confirmed cases to late December 2020 [18]. This study uses a non-linear least squares method to estimate three parameters (*K*, *r*, and *A*) from Equation (3). Additionally, this study analyses the estimated peak time of epidemics or turning points, these are the points where the growth rate of infected cases changes from increasing to decreasing in cases. The study will analyze countries that are thought to have passed (are in the final phase of an epidemic), are ongoing (will be in the final phase of an epidemic), or have not passed (are still in the epidemic phase) turning points (or epidemic peak points) on the *S*-curve.

In this study, a data set of daily and cumulative COVID-19 of confirmed cases in several countries, such as China, Singapore, Saudi Arabia, the Philippines, and Indonesia, will be analyzed using the logistic growth model to validate the accuracy of the method proposed in this study. Since there are countries that are already in the final phase of the epidemic based on the above characteristics, this is likely to provide a good fit between the prediction results and the actual data, so all parameters of the model proposed in this study can be used as validation of this model. Whereas the countries that are still in a state that has not reached the estimated peak time of the epidemic or the turning point, it means that they are still in a phase dominated by the growth rate phase, so this model is expected to be suitable for the countries in this phase. This model will provide a reliable estimation of parameters, not only in the final phase which seems appropriate for the countries in this phase but at other phases during the epidemic. An initial analysis of the epidemic related to the severity of infection and transmission is needed to measure the potential of the COVID-19 pandemic, this is useful to anticipate the increase of the total number of infected cases in the epidemic final.

The unprecedented COVID-19 pandemic has spread very intensely and is still occurring in several countries, so currently, devising the predicting model of transmission of the COVID-19 outbreak is a challenging task. This task is complicated by unreported infected cases, population heterogeneity in infected risk, changes in population behavior, control strategies to contain the infected rate or virus transmission after the initial phase of the epidemic, and stochastic effects.

### 4.1. The Epidemic Case in China

**Figure 1:**
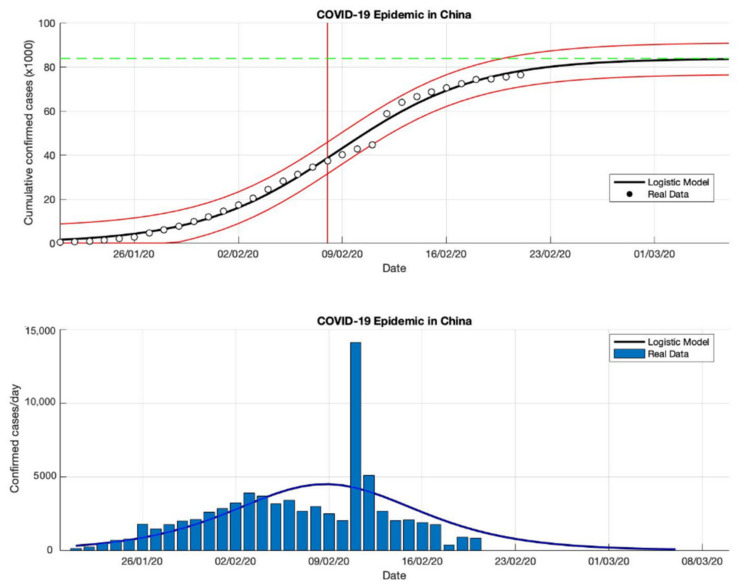
In the above figure, the prediction results of the logistic growth model for cumulative infected cases are compared with the actual data, while the two red lines between the black lines represent a prediction error limit of about 5%. The dashed green line predicts the total size of infected cases in the final phase of the epidemic. The red line intersects the cumulative case curve to predict the turning point or peak point of the epidemic (this line is parallel to the top of the curve from the below figure). In the below figure, the prediction results of this model for daily infected cases in China in the period 21 January to 21 February 2020.

**Figure 2:**
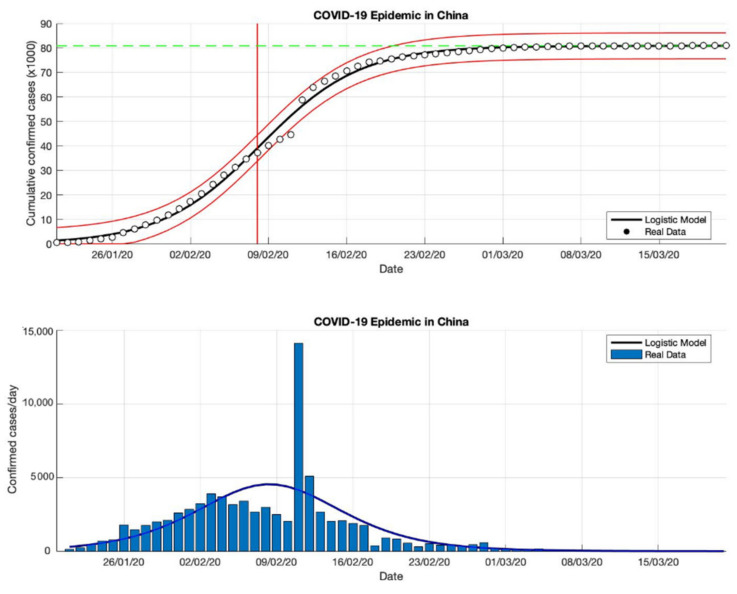
In the above figure, the prediction results of the logistic growth model for cumulative infected cases are compared with the actual data, while the two red lines between the black lines represent a prediction error limit of about 5%. The dashed green line predicts the total size of infected cases in the final phase of the epidemic. The red line intersects the cumulative case curve to predict the turning point or peak point of the epidemic (this line is parallel to the top of the curve from the below figure). In the below figure, the prediction results of this model for daily infected cases in China in the period 21 January to 21 March 2020.

**Figure 3:**
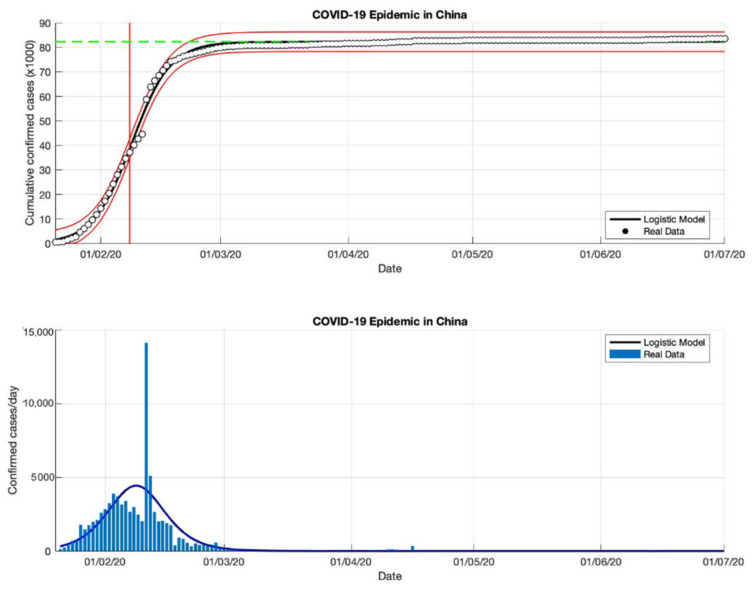
In the above figure, the prediction results of the logistic growth model for cumulative infected cases are compared with the actual data, while the two red lines between the black lines represent a prediction error limit of about 5%. The dashed green line predicts the total size of infected cases in the final phase of the epidemic. The red line intersects the cumulative case curve to predict the turning point or peak point of the epidemic (this line is parallel to the top of the curve from the below figure). In the below figure, the prediction results of this model for daily infected cases in China in the period 21 January to 1 July 2020.

The epidemic case in China was analyzed using data of the cumulative infected cases collected from WHO reports from 21 January 2020, to 21 February 2020 [18]. Additionally, this study extends the analysis using data up to 21 March 2020 [18]. Then, this study also uses data up to the end of June 2020 to expand the epidemic analysis. The non-linear least-squares method is used to analyze the three-time intervals to fit the three parameters (*K*, *r*, and *A*) model of Equation (3). Based on the estimation of the turning point, which is the point where the growth rate begins to decline, as is shown in Figure 1, Figure 2 and Figure 3, the analysis of the prediction results indicates that the epidemic cases in China have passed the estimated turning point on the *S*-curve, and are now in the final phase of the epidemic. A state of this phase provides a good fit for all model parameters from Equation (3).

Based on the prediction results of the three time-intervals of epidemic cases in China, the results of this model show that the total number of the confirmed cases of COVID-19 is around 81,054, 83,802, and 83,488 (these prediction results can be seen in Table 1), while the actual data of the total number of the confirmed cases of COVID-19 is 86,619 [18], it is shown that the actual data is bigger than the predicted results. Furthermore, the estimation results of the epidemic growth rates are 0.260, 0.217, and 0.214 (1/day), it is indicated that the epidemic cases in China have decreased. Then, the estimated results of the turning point or peak time calculated from the first COVID-19 infected cases are 8 February 2020, for the three-time intervals while the estimated end of the transition phase is 26 February 2020. It means that the estimation results from the final phase of the epidemic case in China only lasted about 36 days. The prediction results of this model are within the actual data ranges shown in Figure 1, Figure 2 and Figure 3.

### 4.2. The Epidemic Case in Singapore

Based on the actual data available for the four periods in Singapore, which were taken as input data to the logistic growth model, the prediction results of the final size of the COVID-19 pandemic using the logistic growth model were around 31,835, 42,432, 57,639, and 58,422, respectively. These prediction results can be seen in Table 2. Meanwhile, the estimation results of the turning point or peak time of the epidemic occurred on 30 April, 7 May, 22 May, and 25 May, respectively. The peak time is obtained around 123 days since the first confirmed case of COVID-19. It can be seen that the transition phase towards the final phase of the epidemic will be at the end of May 2020. This is also confirmed by the prediction results of the growth rate which has started to decline, estimated results of around 0.115, 0.081, 0.046, and 0.042 (1/day), respectively. The actual number of cases infected with COVID-19 was 58,432 cases at the end of December 2020, while the prediction result was around 58,422.

**Figure 4:**
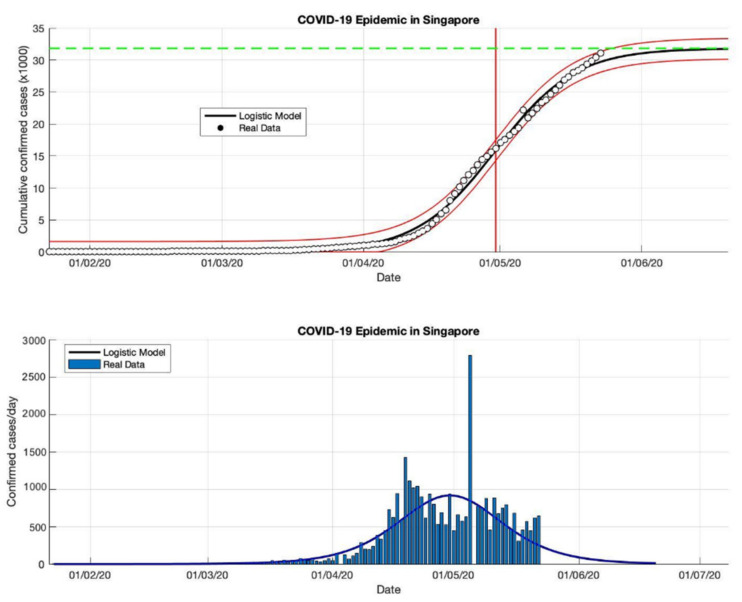
In the above figure, the prediction results of the logistic growth model for cumulative infected cases are compared with the actual data, while the two red lines between the black lines represent a prediction error limit of about 5%. The dashed green line predicts the total size of infected cases in the final phase of the epidemic. The red line intersects the cumulative case curve to predict the turning point or peak point of the epidemic (this line is parallel to the top of the curve from the below figure). In the below figure, the prediction results of this model for daily infected cases in Singapore in the period 23 January to 23 May 2020.

**Figure 5:**
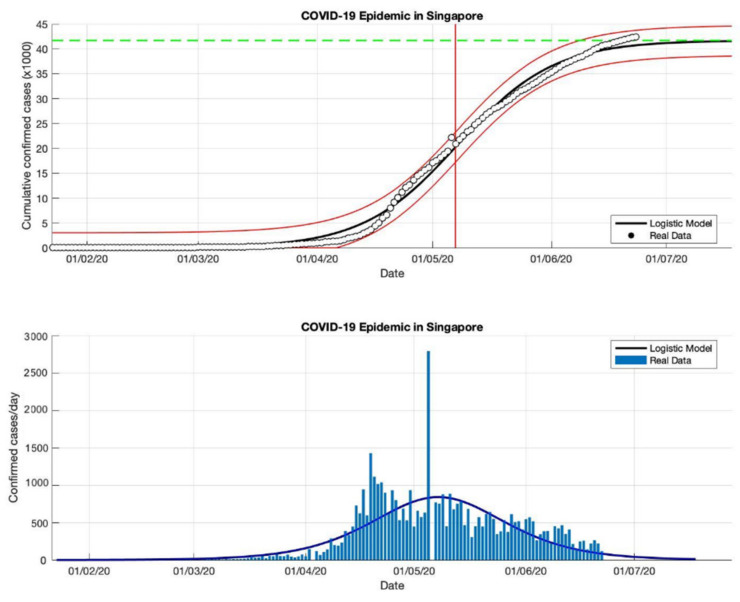
In the above figure, the prediction results of the logistic growth model for cumulative infected cases are compared with the actual data, while the two red lines between the black lines represent a prediction error limit of about 5%. The dashed green line predicts the total size of infected cases in the final phase of the epidemic. The red line intersects the cumulative case curve to predict the turning point or peak point of the epidemic (this line is parallel to the top of the curve from the below figure). In the below figure, the prediction results of this model for daily infected cases in Singapore in the period 23 January to 23 June 2020.

**Figure 6:**
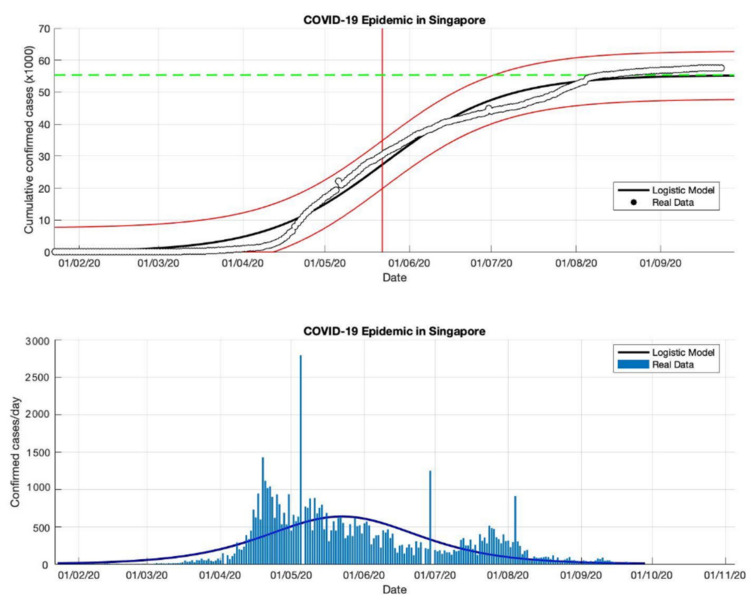
In the above figure, the prediction results of the logistic growth model for cumulative infected cases are compared with the actual data, while the two red lines between the black lines represent a prediction error limit of about 5%. The dashed green line predicts the total size of infected cases in the final phase of the epidemic. The red line intersects the cumulative case curve to predict the turning point or peak point of the epidemic (this line is parallel to the top of the curve from the below figure). In the below figure, the prediction results of this model for daily infected cases in Singapore in the period 23 January to 23 September 2020.

**Figure 7:**
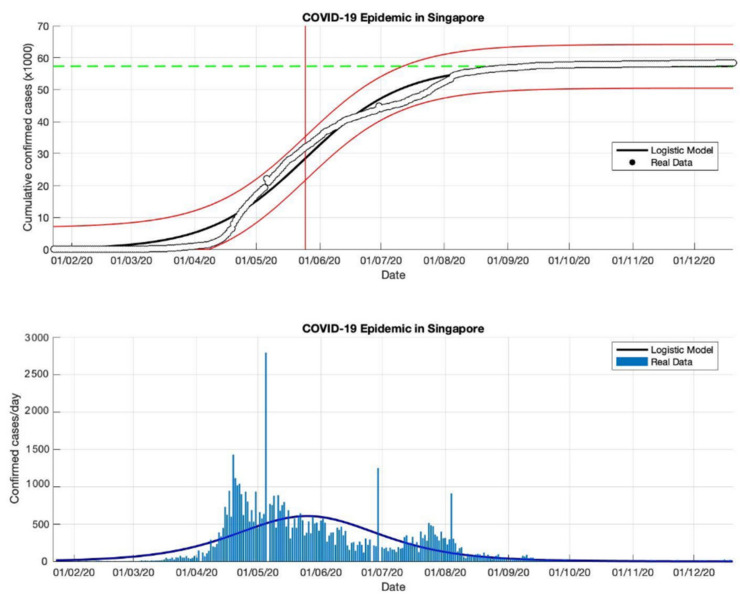
In the above figure, the prediction results of the logistic growth model for cumulative infected cases are compared with the actual data, while the two red lines between the black lines represent a prediction error limit of about 5%. The dashed green line predicts the total size of infected cases in the final phase of the epidemic. The red line intersects the cumulative case curve to predict the turning point or peak point of the epidemic (this line is parallel to the top of the curve from the below figure). In the below figure, the prediction results of this model for daily infected cases in Singapore in the period 23 January to 23 December 2020.

In the first and second periods, modeling was still not complicated (as shown in Figure 4 and Figure 5), however, modeling uses actual data in Singapore is a much more complicated process. Hence, Figure 6 and Figure 7 show some of the peaks in the time series of new daily cases of COVID-19 in Singapore. As a result, the logistic growth model built using data from the first period, 23 January to 23 May 2020, failed to reach a new peak after that date. When this model is used to estimate using more actual data and the time limit set is extended to 23 June and 23 September 2020, respectively, the prediction results show how this logistic model has evolved and adapted better to the actual data.

Although this model still fails to provide an estimation of outbreaks in the early stages, because this model only produces a maximum increase in single cases or can only predict the first wave, however, the analysis carried out on the parameter *r* and this study found that each period shows a decrease in the growth rate of the COVID19 infected cases. This study suspects that this is due to better public health policies, better public behavior, or better measures to prevent the spread of the virus. This study also analyzed that the variation of the *R*^2^ value of the non-linear curve in each period was different, this shows that there is a better serial correlation for each period.

### 4.3. The Epidemic Case in Saudi Arabia

The logistic growth model approach has been used to predict the cumulative confirmed cases in Saudi Arabia from the start of the epidemic on 2 March to 2 May 2020. The first period for predicting short-term forecasts was carried out for preventive measures in Saudi Arabia. The parameter estimation results are presented in Table 3. Furthermore, other short-term estimates of cumulative confirmed cases in Saudi Arabia are also presented in Table 3. The prediction results of cumulative confirmed cases of COVID-19 infected in Saudi Arabia are arranged into three periods, starting from 2 March to 23 December 2020. Based on the estimated turning point or peak time of the epidemic in Saudi Arabia is at the end of June or around 26 June 2020, it means that it has been around 116 days since the first confirmed case of COVID-19. The estimation results of the cumulative number of COVID-19 confirmed cases in Saudi Arabia is around 361,010, while the actual data obtained from the WHO source is around 361,178 [18]. It shows that the results obtained from the prediction results are smaller than the actual data. The growth rates of infected cases obtained by this model also decreased: 0.118, 0.053, 0.048, and 0.041 (1/day), respectively.

**Figure 8:**
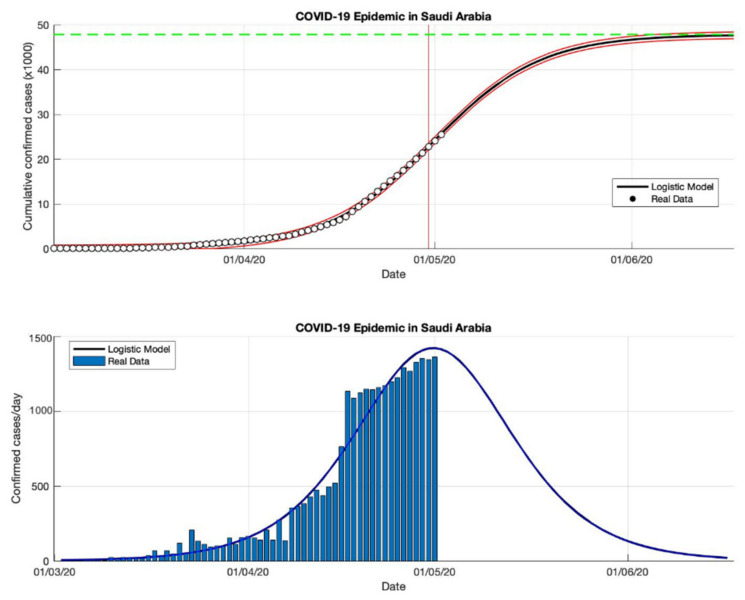
In the above figure, the prediction results of the logistic growth model for cumulative infected cases are compared with the actual data, while the two red lines between the black lines represent a prediction error limit of about 5%. The dashed green line predicts the total size of infected cases in the final phase of the epidemic. The red line intersects the cumulative case curve to predict the turning point or peak point of the epidemic (this line is parallel to the top of the curve from the below figure). In the below figure, the prediction results of this model for daily infected cases in Saudi Arabia in the period 2 March to 2 May 2020.

**Figure 9:**
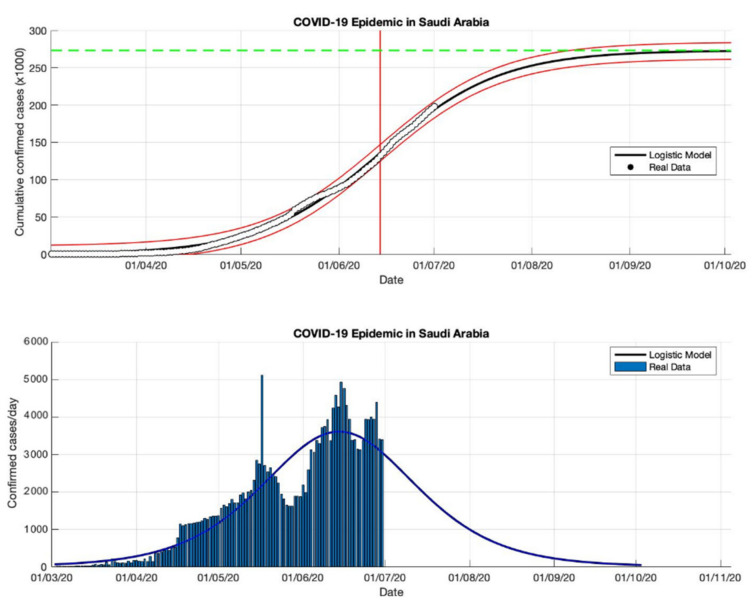
In the above figure, the prediction results of the logistic growth model for cumulative infected cases are compared with the actual data, while the two red lines between the black lines represent a prediction error limit of about 5%. The dashed green line predicts the total size of infected cases in the final phase of the epidemic. The red line intersects the cumulative case curve to predict the turning point or peak point of the epidemic (this line is parallel to the top of the curve from the below figure). In the below figure, the prediction results of this model for daily infected cases in Saudi Arabia in the period 2 March to 2 July 2020.

**Figure 10:**
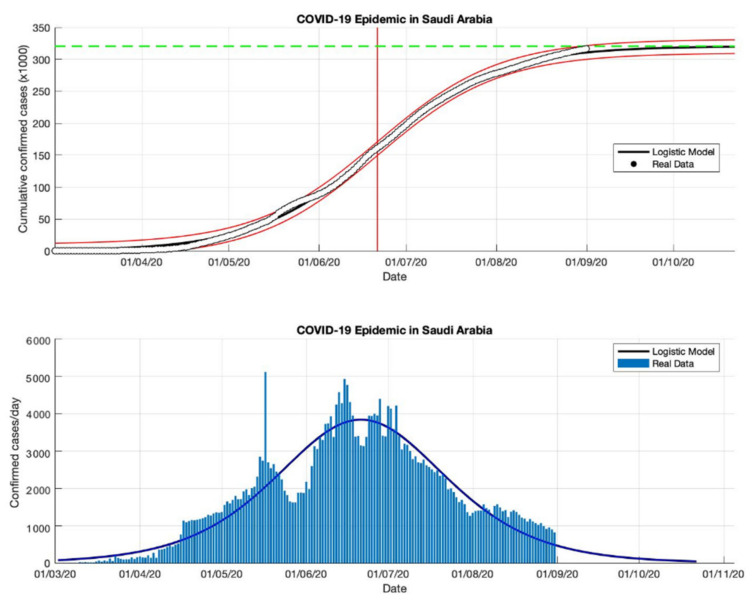
In the above figure, the prediction results of the logistic growth model for cumulative infected cases are compared with the actual data, while the two red lines between the black lines represent a prediction error limit of about 5%. The dashed green line predicts the total size of infected cases in the final phase of the epidemic. The red line intersects the cumulative case curve to predict the turning point or peak point of the epidemic (this line is parallel to the top of the curve from the below figure). In the below figure, the prediction results of this model for daily infected cases in Saudi Arabia in the period 2 March to 2 September 2020.

**Figure 11:**
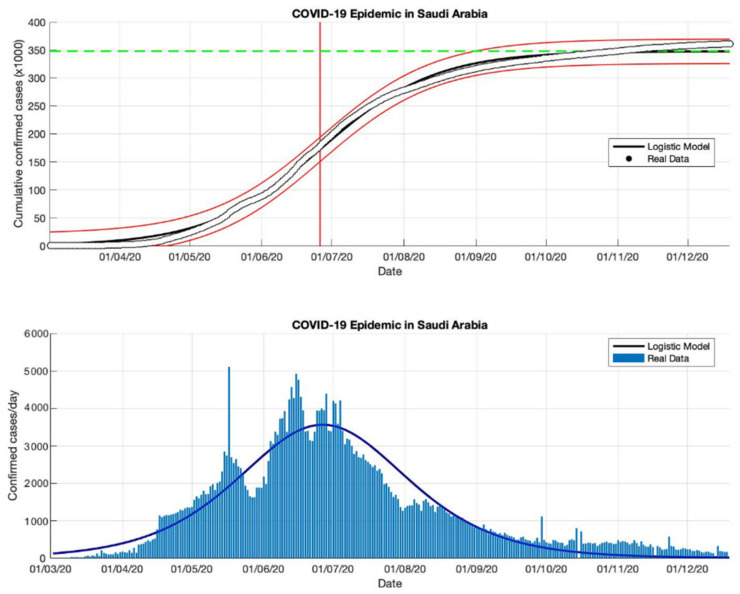
In the above figure, the prediction results of the logistic growth model for cumulative infected cases are compared with the actual data, while the two red lines between the black lines represent a prediction error limit of about 5%. The dashed green line predicts the total size of infected cases in the final phase of the epidemic. The red line intersects the cumulative case curve to predict the turning point or peak point of the epidemic (this line is parallel to the top of the curve from the below figure). In the below figure, the prediction results of this model for daily infected cases in Saudi Arabia in the period 2 March to 23 December 2020.

A data-based prediction model that relies on time series data shows that the simulation results look more optimistic if the actual data does not fluctuate. This can be seen from the peak number of virus infections leading to its peak time (see Figure 8, Figure 9, Figure 10 and Figure 11) and the transition of the epidemic phase from 14 June to 26 June 2020. The prediction results based on current data show that the epidemic continues to decline in Saudi Arabia, however, we need further predictions to ensure this when more data become available.

In general, an estimation of the parameters of an epidemiological model is always difficult and requires strong assumptions to obtain values that are consistent and robust throughout the epidemic. The new estimate developed in this study, a phenomenological model of infection growth based on the classic Verhulst model derives explicit formulas for the values of important parameters, such as intrinsic growth rate and the accumulated number of infected cases, which provides a near-perfect estimate of the observed dynamics. 

The use of this phenomenological model can also be considered as a way of smoothing data because errors in predicting the newly infected cases can occur if tracking of the newly infected cases is rarely carried out regularly so that the reporting of the new cases is delayed for several weeks and at certain times it will be adding data excessively than the actual calculation. Since the number of new cases observed may still be underestimated so the number of new cases that are not reported will always be a difficult problem. The actual data is sometimes reduced due to reasons of poor diagnosis or lack of detection tools, or reasons of country policies.

### 4.4. The Epidemic Case in the Philippines

Based on an estimation of this model, at least 459,784 infected cases of COVID-19 will be reached by the final time the epidemic in the Philippines (as shown in Table 4). This number is slightly smaller than the number of infections reported by WHO [18] of around 461,505. However, the estimated daily growth rates for COVID-19 in the Philippines are still fluctuating, namely 0.118, 0.032, 0.038, and 0.033 (1/day), respectively. This means indicates that the total number of COVID-19 cases in the Philippines will still double if there are no anti-epidemic action measures. The estimation of the turning point or peak time for daily new infected cases is also not yet on a specific date. In this study, the predicted peak time will be around 5 September 2020, or about 184 days from the start of the epidemic on 5 March 2020. However, based on the predicted growth rate, these prediction results indicate that changes in COVID-19 cases before and after the predicted turning point on 5 September will follow the same pattern.

**Figure 12:**
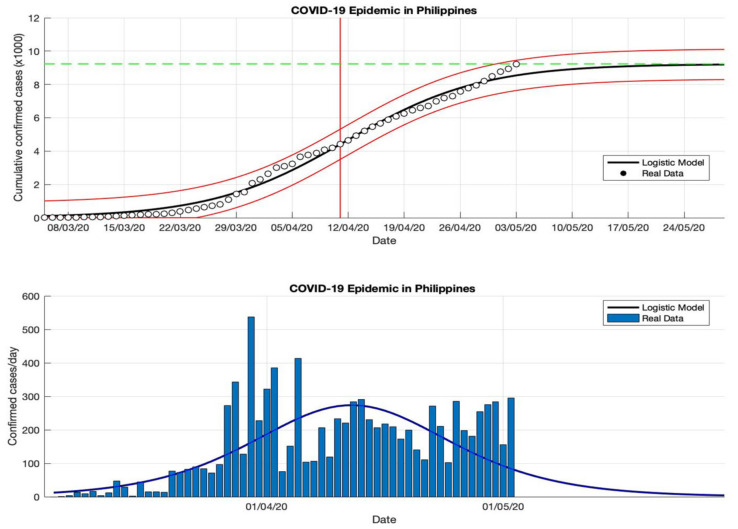
In the above figure, the prediction results of the logistic growth model for cumulative infected cases are compared with the actual data, while the two red lines between the black lines represent a prediction error limit of about 5%. The dashed green line predicts the total size of infected cases in the final phase of the epidemic. The red line intersects the cumulative case curve to predict the turning point or peak point of the epidemic (this line is parallel to the top of the curve from the below figure). In the below figure, the prediction results of this model for daily infected cases in the Philippines in the period 5 March to 5 May 2020.

**Figure 13:**
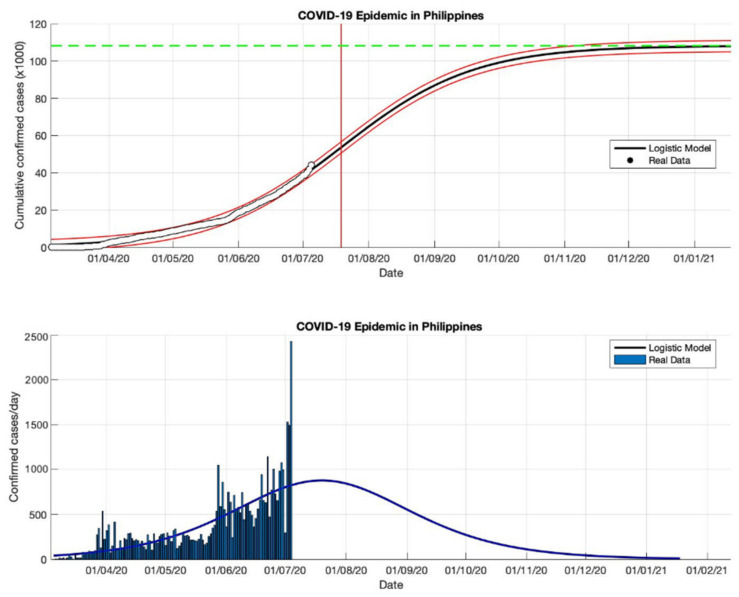
In the above figure, the prediction results of the logistic growth model for cumulative infected cases are compared with the actual data, while the two red lines between the black lines represent a prediction error limit of about 5%. The dashed green line predicts the total size of infected cases in the final phase of the epidemic. The red line intersects the cumulative case curve to predict the turning point or peak point of the epidemic (this line is parallel to the top of the curve from the below figure). In the below figure, the prediction results of this model for daily infected cases in the Philippines in the period 5 March to 5 July 2020.

**Figure 14:**
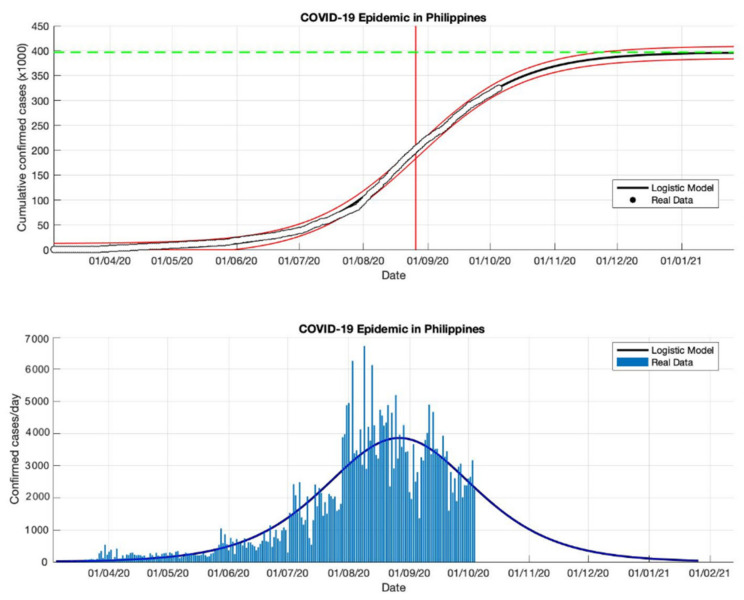
In the above figure, the prediction results of the logistic growth model for cumulative infected cases are compared with the actual data, while the two red lines between the black lines represent a prediction error limit of about 5%. The dashed green line predicts the total size of infected cases in the final phase of the epidemic. The red line intersects the cumulative case curve to predict the turning point or peak point of the epidemic (this line is parallel to the top of the curve from the below figure). In the below figure, the prediction results of this model for daily infected cases in the Philippines in the period 5 March to 5 October 2020.

**Figure 15:**
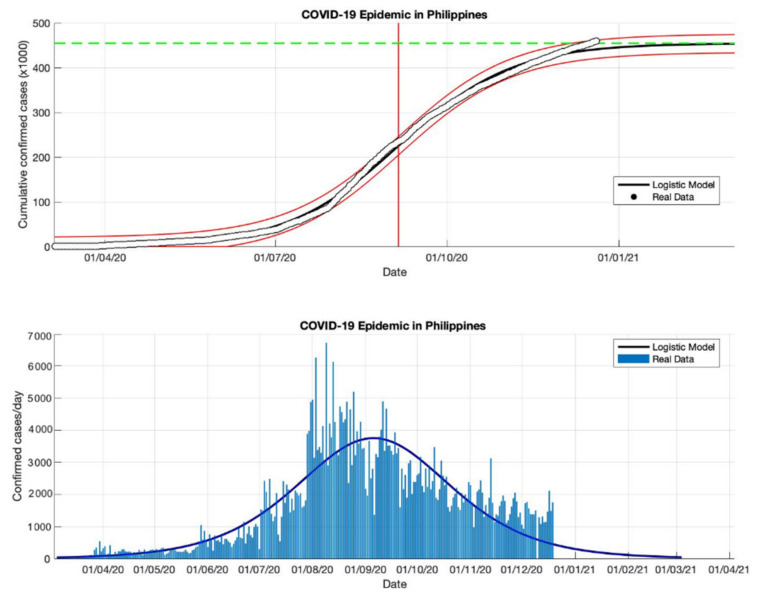
In the above figure, the prediction results of the logistic growth model for cumulative infected cases are compared with the actual data, while the two red lines between the black lines represent a prediction error limit of about 5%. The dashed green line predicts the total size of infected cases in the final phase of the epidemic. The red line intersects the cumulative case curve to predict the turning point or peak point of the epidemic (this line is parallel to the top of the curve from the below figure). In the below figure, the prediction results of this model for daily infected cases in the Philippines in the period 5 March to 23 December 2020.

Equations (13)–(15) will adjust to the parameters estimated in this model based on actual data in each country, then these parameters are used as input in the prediction model. Based on the epidemic case in the Philippines (see Figure 12, Figure 13, Figure 14 and Figure 15), the non-linear regression method shows consistent results, this is consistent with actual data based on the *R*^2^ value for each epidemic period. It is also because the actual data in the corresponding time series are on a non-linear curve, i.e., the peak period of the outbreak with most cases and the period after the peak covering the rightmost tail with some cases. This study identifies that if the prediction results are close to new cases that are sporadic or fluctuating, the *R*^2^ value is still stable, even though the *R*^2^ value is in the 90–100% interval but the value is not below 90%. Consequently, the probability of the *R*^2^ value in each part of the period differs from the non-linear curve, but it may still be possible to use it to compare the suitability in each part of the period.

### 4.5. The Epidemic Case in Indonesia

**Figure 16:**
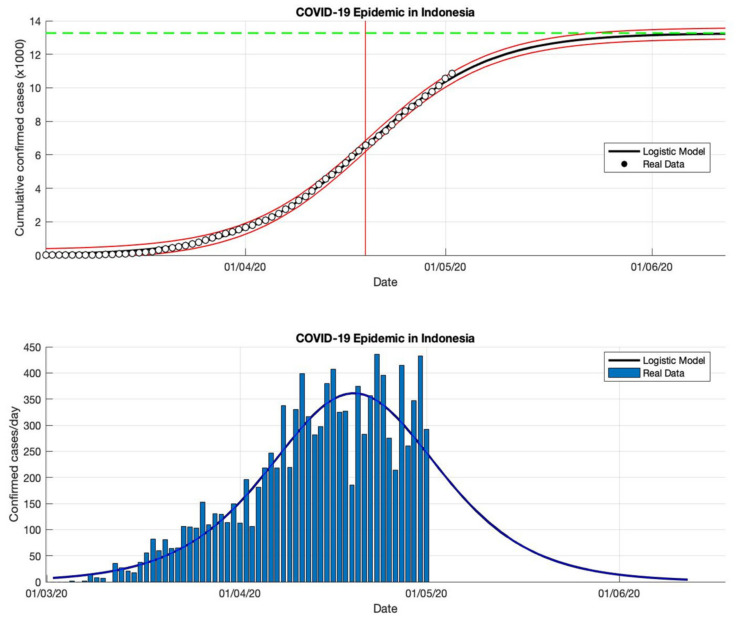
In the above figure, the prediction results of the logistic growth model for cumulative infected cases are compared with the actual data, while the two red lines between the black lines represent a prediction error limit of about 5%. The dashed green line predicts the total size of infected cases in the final phase of the epidemic. The red line intersects the cumulative case curve to predict the turning point or peak point of the epidemic (this line is parallel to the top of the curve from the below figure). In the below figure, the prediction results of this model for daily infected cases in Indonesia in the period 2 March to 2 May 2020.

**Figure 17:**
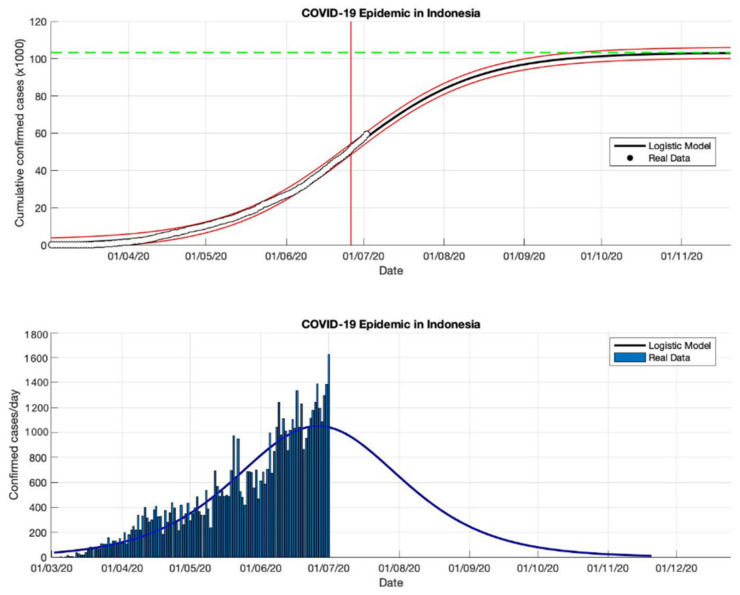
In the above figure, the prediction results of the logistic growth model for cumulative infected cases are compared with the actual data, while the two red lines between the black lines represent a prediction error limit of about 5%. The dashed green line predicts the total size of infected cases in the final phase of the epidemic. The red line intersects the cumulative case curve to predict the turning point or peak point of the epidemic (this line is parallel to the top of the curve from the below figure). In the below figure, the prediction results of this model for daily infected cases in Indonesia in the period 2 March to 2 July 2020.

**Figure 18:**
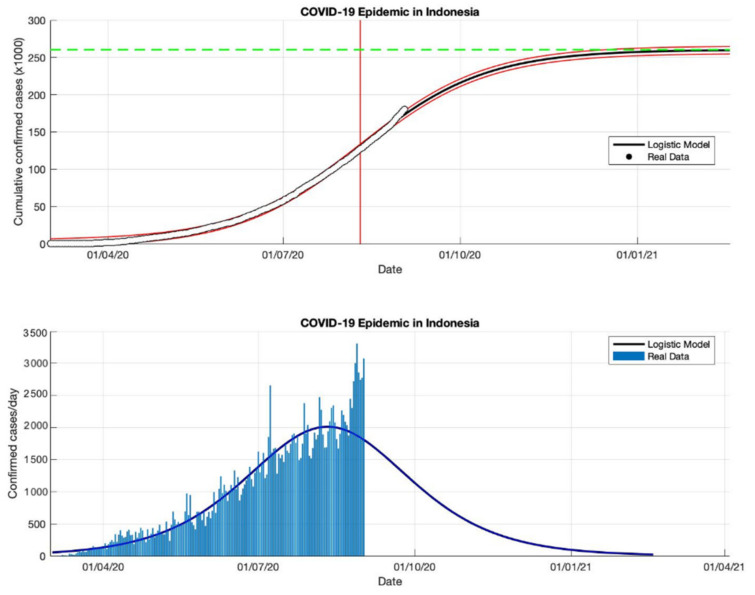
In the above figure, the prediction results of the logistic growth model for cumulative infected cases are compared with the actual data, while the two red lines between the black lines represent a prediction error limit of about 5%. The dashed green line predicts the total size of infected cases in the final phase of the epidemic. The red line intersects the cumulative case curve to predict the turning point or peak point of the epidemic (this line is parallel to the top of the curve from the below figure). In the below figure, the prediction results of this model for daily infected cases in Indonesia in the period 2 March to 2 September 2020.

**Figure 19:**
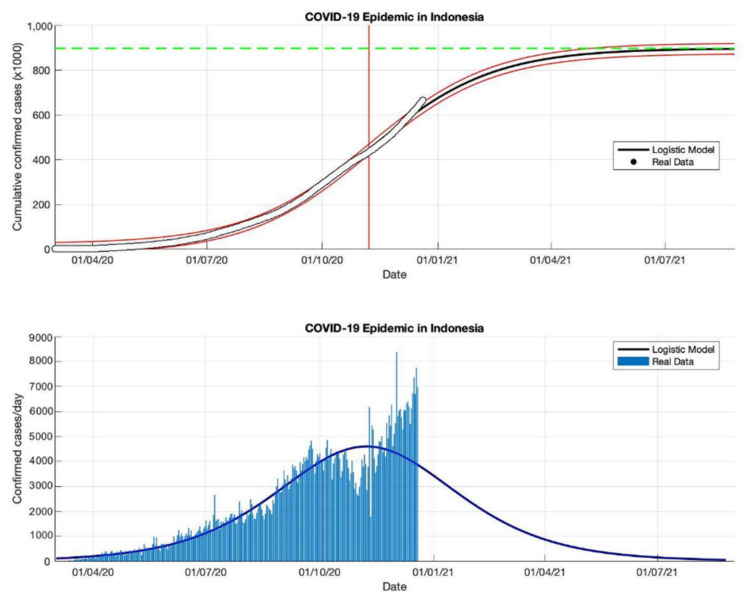
In the above figure, the prediction results of the logistic growth model for cumulative infected cases are compared with the actual data, while the two red lines between the black lines represent a prediction error limit of about 5%. The dashed green line predicts the total size of infected cases in the final phase of the epidemic. The red line intersects the cumulative case curve to predict the turning point or peak point of the epidemic (this line is parallel to the top of the curve from the below figure). In the below figure, the prediction results of this model for daily infected cases in Indonesia in the period 2 March to 23 December 2020.

This study proposes an epidemic model as a function of time-series data to fit a logistic growth curve with the number of cumulative infected cases over the time series. This model uses a phenomenological approach that is not complex, but it can describe the approach from epidemic data that is changing rapidly and allows the results of this approach to be fairly accurate predictions. This prediction logistic growth model is applied to actual data from cumulative or daily infected cases in Indonesia. The reason is, Indonesia is still a country with active transmission, based on data from WHO [18] which still notes that the estimate of confirmed positive cases continues to increase. It is also indicated by the high transmission of COVID-19, and the outbreak which is still far from over (see Table 5 and Figure 16, Figure 17, Figure 18 and Figure 19).

In each period, the actual data was taken when the first COVID-19 cases appeared, starting 2 March 2020, then the actual data were collected on 2 May, 2 July, 2 September, and 23 December 2020. The prediction results from this model produced estimated cumulative infections, namely around 13,268, 103,283, 260,316, and 896,504 in the final phase of the COVID-19 pandemic (as shown in Table 5). This model also estimates the per capita growth rates of the infected population are 0.109, 0.041, 0.047, and 0.049. The prediction results obtained for the turning point or peak time of the epidemic have not yet reached a certain time. Each period has a determinant coefficient value (*R*^2^) above 95% which shows the prediction results are following the actual data.

Although this model is phenomenological by mimicking the spread of the epidemic, the prediction of daily infected cases and the prediction of the cumulative case size at the final epidemic can be used as a preventive measure, so that controlling the spread of this outbreak as a preventive measure can be carried out based on the prediction of the COVID-19 pandemic. Any deviation from the prediction curve can indicate that the epidemic may have gotten out of control, which could be if the actual data appear distorted and do not follow a typical logistic curve. In the earliest period, based on actual data reported, the number of confirmed COVID-19 cases grew slowly during the spread of the epidemic. Then, the next stage of growth is exponential growth. When saturation begins to emerge, growth slows down to a linear rate, and during the final phase of the epidemic, growth will stop. The epidemic process can be predicted well by this model.

## 5. Conclusions

In this study, a simple equation of the logistic growth model with the three parameters estimated using the non-linear least-squares method has been proposed to analyze cases of the COVID-19 pandemic in several countries. This model has provided knowledge about a current epidemiological study and can be used as a reference in the future. This study is also able to provide a qualitative estimation of COVID-19 infected cases reviewed in terms of this model accuracy is based on the *R*^2^ value of the non-linear curve formed. Although, this model still does not provide a quantitative estimation of COVID-19 infected cases (there are differences in the daily and the cumulative number of COVID-19 infected cases). However, this model provides good prediction results in terms of the patterns of increases and decreases in the number of daily and cumulative infected cases of COVID-19.

This study has conducted an analysis based on the value of the parameter *r*, there were differences in the intrinsic growth rate of cases of the COVID-19 infected. It can be due to the possibility of differences in public health policymaking in restraining the transmission process, such as community behavior (wearing masks, wearing hand sanitizers, and physical distancing), handling infected patients by medical personnel, methods of diagnosing newly infected cases, and others. Therefore, this model has the potential to contribute to formulating public health policies to suppress the transmission process better and to prevent the COVID-19 pandemic in each region.

However, all predictions of this model are based on the assumption that there is always one peak or a maximum value of the epidemic on the curve of daily infected cases modeled by the complete logistic curve. Whereas the epidemic curve is real, there are likely some small peaks during the epidemic due to differences in epidemic prevention measures, mainly tracking and detection (diagnosis) of individuals suspected of being infected in each country.

In this study, the logistic growth model has been used to predict the COVID-19 pandemic. The prediction procedure of this model is easy to implement and can be run with commercially available software or free access software (not commercial). This model can also analyze the severity during the COVID-19 pandemic. It may be that the actual available data is limited or incomplete during the COVID-19 pandemic, but initial input from this simple model requires only actual data that can be easily obtained in an epidemic situation. Therefore, this model offers the best opportunity for practical solutions to identify and predict and then control the epidemic on time. Although this simple model does not provide accurate numerical predictions, it can be used to predict qualitatively or in other terms quantitatively, which is still quite rough. Therefore, the prediction accuracy of this model is highly dependent on the absence of stochastic events occurring during (the remaining days) of the epidemic as this could significantly change the course of this epidemic phase.

## Figures and Tables

**Table 1:** The results of estimated parameters in the logistic growth model for various periods that are compared by daily cases data of COVID-19 infected in China based on data compiled by WHO [18].

Period 2020	Turning Point *t_p_*	Growth Rate *r*	The Estimated Size of Cases *K*	Parameter *A*	Value *R*^2^
21 January–21 February	8 February 2020	0.260	81,054	411,141	0.993
21 January–21 March	8 February 2020	0.217	83,802	465,153	0.993
21 January–1 July	8 February 2020	0.214	83,488	267,044	0.997

**Table 2:** The estimation results of parameters in the logistic growth model for various periods from daily cases data of COVID-19 infected in Singapore based on data compiled by WHO [18].

Period 2020	Turning Point *t_p_*	Growth Rate *r*	The Estimated Size of Cases *K*	Parameter *A*	Value *R*^2^
23 January–23 May	30 April 2020	0.115	31,835	47,179	0.990
23 January–23 June	7 May 2020	0.081	42,432	57,981	0.996
23 January–23 September	22 May 2020	0.046	57,639	76,005	0.988
23 January–23 December	25 May 2020	0.042	58,422	72,928	0.991

**Table 3:** The estimation results of parameters in the logistic growth model for various periods from daily cases data of COVID-19 infected in Saudi Arabia based on data compiled by WHO [18].

Period 2020	Turning Point *t_p_*	Growth Rate *r*	The Estimated Size of Cases *K*	Parameter *A*	Value *R*^2^
2 March–2 May	30 April 2020	0.118	47,795	52,847	0.999
2 March–2 July	14 June 2020	0.053	273,139	326,863	0.996
2 March–2 September	21 June 2020	0.048	320,760	467,156	0.999
2 March–23 December	26 June 2020	0.041	361,010	480,959	0.997

**Table 4:** The estimation results of parameters in the logistic growth model for various periods from daily cases data of COVID-19 infected in the Philippines based on data compiled by WHO [18].

Period 2020	Turning Point *t_p_*	Growth Rate *r*	The Estimated Size of Cases *K*	Parameter *A*	Value *R*^2^
5 March–5 May	11 April 2020	0.118	9,230	3,711	0.991
5 March–5 July	19 July 2020	0.032	108,239	13,100	0.993
5 March–5 October	26 August 2020	0.038	396,985	83,389	0.998
5 March–23 December	5 September 2020	0.033	459,784	113,105	0.999

**Table 5:** The estimation results of parameters in the logistic growth model for various periods from daily cases data of COVID-19 infected in Indonesia based on data compiled by WHO [18].

Period 2020	Turning Point *t_p_*	Growth Rate *r*	The Estimated Size of Cases *K*	Parameter *A*	Value *R*^2^
2 March–2 May	19 April 2020	0.109	13,268	10,830	0.989
2 March–2 July	26 June 2020	0.041	103,283	46,139	0.996
2 March–2 September	10 August 2020	0.047	260,316	124,384	0.997
2 March–23 December	7 November 2020	0.049	896,504	412,832	0.999

## Data Availability

Actual data comes from the World Health Organization (WHO) Coronavirus (COVID-19) dashboard which is available online: https://covid19.who.int// and accessed on 23 December 2020.
